# NOW SHOWING: THE USE OF FILMS IN ADVANCED GERONTOLOGY COURSES

**DOI:** 10.1093/geroni/igad104.2119

**Published:** 2023-12-21

**Authors:** Jessica VanderWerf, Phyllis Greenberg, Rona Karasik

**Affiliations:** University of South Florida, Tampa, Florida, United States; St. Cloud State University, St. Cloud, Minnesota, United States; Saint Cloud State University, Saint Cloud, Minnesota, United States

## Abstract

While the use of films to teach about aging and older adults is not new, current movies and the students who view them are ever-changing. So too are the learning goals and practices for incorporating media into the advanced gerontological classroom. The current undertaking considers the unique aspects of employing movies (including documentaries and international films) and builds on previous works (Cohen-Shalev & Marcus, 2007; Karasik & Hamon, 2014; Markson & Taylor 1993) with an eye toward the more advanced pedagogical goals of upper level undergraduate and graduate courses. This poster focuses on challenges associated with including film in both traditional and emerging classroom delivery formats (e.g., face to face, hybrid, on-line). A matrix of current aging-themed films is provided, along with strategies for successful applications in upper level and graduate courses. Karasik and Hamon (2014) explored the use of popular film in introductory gerontology courses, creating a detailed matrix of films spanning 1951 - 2012. In addition to film descriptions and ratings, the matrix identified content in the following topical areas in (ageism/stereotypes; cognitive impairment; death & dying; diversity; family relationships; health & wellness; sexuality & intimacy; and work & retirement). The current presentation builds on Karasik & Hamon’s work by developing a companion matrix of films including feature films from 2012- 2023 , as well as documentaries and international films across the same time period. In addition to the newly created companion matrix, there are examples of activities and assignments that incorporate movies into the gerontological classroom.

